# Brain Workers

**Published:** 1889-05

**Authors:** 


### Brain-Workers.

The *Medical Age* says that the most frequent fault of the brain-worker is excessive application to work. "The most intense and fatiguing of toils is pursued almost uninterruptedly, food is neglected, and the claims of exercise and sleep are but imperfectly admitted. Two hours' exercise in the open air, daily, is propably a minimum, and might prudently be exceeded. The brain-worker must live sparingly rather than luxuriantly; he must prefer the lighter classes of food to the heavier, and he must be very prudent in the use of alcohol. Tobacco and tea are apt to be favorites with him, and their immoderate use may require to be guarded against. It is a nice question whether he needs more or less sleep than other men. Many men of genius are light sleepers; probably in some cases a misfortune; but there seems some ground for the notion that more than a moderate indulgence in sleep is unfavorable to successful mental effort."

A commentator upon the above remarks says that he can not fully agree with them. Mental effort, he says, and we agree with him, causes waste of tissue-elements quite as much as bodily exertion, and this demands a full supply of food. What with dyspepsia and absence of appetite, the results of deficient exercise, and the influence of preconceived ideas as to the use or disuse of special articles of food, the brain-worker is very apt to receive too little nutriment to make up for the waste. Especially is this the case when he, unconsciously, perhaps, replaces food by the use of tobacco, tea, alcohol, or opium.

Some advise to go supperless to bed. This is a wrong notion. It is a fruitful source of insomnia a neurasthenia. The brain becomes exhausted by its evening work, and demands rest and refreshment of its wasted tissues; not by indigestible salads and "fried abominations," but by some nutritious, easily-digested and assimilated articles. A bowl of stale bread and milk, of rice or some other farinaceous food, with milk or hot soup, would be more to the purpose. Any of these would insure a sound night's sleep, from which the man would awaken refreshed.

The man who desires to realize from his stock of brains and from his bodily vigor to the utmost possible amount must regulate his life by rigid precepts. He must eat food suited to his needs and the powers of his stomach; he must eschew artificial stimulants; he must keep the Sabbath, at least physiologically. He *must* keep his mind free from financial worry; he must steel his heart against the allurements of Bacchus and Venus.

